# Functional Adaptation in the Brain Habenulo–Mesencephalic Pathway During Cannabinoid Withdrawal

**DOI:** 10.3390/cells13211809

**Published:** 2024-11-01

**Authors:** Sonia Aroni, Claudia Sagheddu, Marco Pistis, Anna Lisa Muntoni

**Affiliations:** 1 Department of Biomedical Sciences, Division of Neuroscience and Clinical Pharmacology, University of Cagliari, Cittadella Universitaria di Monserrato, I-09042 Monserrato, Italy; sonia.aroni@unica.it (S.A.); claudiasagheddu@unica.it (C.S.); mpistis@unica.it (M.P.); 2 Neuroscience Institute, Section of Cagliari, National Research Council of Italy, Cittadella Universitaria di Monserrato, I-09042 Cagliari, Italy; 3 Unit of Clinical Pharmacology, University Hospital, I-09123 Cagliari, Italy

**Keywords:** lateral habenula, rostromedial tegmental nucleus, ventral tegmental area, dopamine, GABA, Δ^9^-tetrahydrocannabinol, in vivo electrophysiology, behavior, substance use disorders

## Abstract

The mesolimbic reward system originating from dopamine neurons in the ventral tegmental area (VTA) of the midbrain shows a profound reduction in function during cannabinoid withdrawal. This condition may underlie aversive states that lead to compulsive drug seeking and relapse. The lateral habenula (LHb) exerts negative control over the VTA via the GABA rostromedial tegmental nucleus (RMTg), representing a potential convergence point for drug-induced opponent processes. We hypothesized that the LHb–RMTg pathway might be causally involved in the hypodopaminergic state during cannabinoid withdrawal. To induce Δ^9^-tetrahydrocannabinol (THC) dependence, adult male Sprague–Dawley rats were treated with THC (15 mg/kg, i.p.) twice daily for 6.5–7 days. Administration of the cannabinoid antagonist rimonabant (5 mg/kg, i.p.) precipitated a robust behavioral withdrawal syndrome, while abrupt THC suspension caused milder signs of abstinence. Extracellular single unit recordings confirmed a marked decrease in the discharge frequency and burst firing of VTA dopamine neurons during THC withdrawal. The duration of RMTg-evoked inhibition was longer in THC withdrawn rats. Additionally, the spontaneous activity of RMTg neurons and of LHb neurons was strongly depressed during cannabinoid withdrawal. These findings support the hypothesis that functional changes in the habenulo–mesencephalic circuit are implicated in the mechanisms underlying substance use disorders.

## 1. Introduction

Cannabis is one of the most commonly used illicit drugs and its consumption is constantly growing [1]. Despite that public “cannabis use risk perception” has lowered in the past decade [1] and the political debate is shifting toward legalization [2], its derivatives have been fully recognized as addictive, with cannabis misuse and dependence integrated among other substance use disorders in the Diagnostic and Statistical Manual of Mental Disorders (DSM-5) [3,4]. Notably, the widespread use of cannabis has become complicated not only by recent changes in legislation and increased availability for therapeutic purposes [5] but also by increased content up to 90% [6,7] of Δ^9^-tetrahydrocannabinol (THC), the main psychoactive component. Licensed pharmacological treatments for cannabis use disorder(s) (CUDs) are currently not available, although the number of treatment-seeking patients is rising [3,8,9]. Potential medications aim to reduce or extinguish withdrawal symptoms and drug craving in order to prevent relapse, and then cease the cycling/chronic nature of addiction [8,10]. According to the DSM-5, the occurrence of a characteristic withdrawal syndrome, the major symptoms of which are irritability, anxiety, sleeping problems, depressed mood, and pain, is a criterion for diagnosis of CUD [4,11]. During withdrawal, allostatic changes and maladaptive remodeling in the brain are associated with an aversive emotional state [11,12,13,14]. The avoidance of discomfort is considered to contribute to the negative reinforcement that leads toward compulsive drug consumption and relapse after abstinence [15,16]. Hence, understanding the neurobiology of cannabis withdrawal is key for research and treatment in the field of addiction.

The mesolimbic dopamine system, central in the brain reward circuit, is mainly comprised of ventral tegmental area (VTA) dopamine neurons that project to the *nucleus accumbens* (NAc). It was reported that these cells display reduced activity during acute and prolonged withdrawal after chronic intake of major drugs of abuse, including cannabinoids [17]. In particular, we previously showed that acute withdrawal after chronic THC exposure results in a decreased firing rate and bursting activity of VTA dopamine neurons [18]. Accordingly, extracellular dopamine levels are reduced in the NAc [19]. Such a hypodopaminergic state, which also characterizes the brain of chronic cannabis users [6,20,21,22], is independent of physical manifestations of drug abstinence [17,18], and it is thought to contribute to withdrawal-induced negative affects. Yet, the underlying mechanisms are little explored.

Firing activity of VTA dopamine neurons is finely tuned by different inputs [23,24,25]. Among these, the lateral habenula–rostromedial tegmental nucleus (LHb–RMTg) circuit has gained prominence. Activation of both the LHb and RMTg is known to inhibit the majority of dopamine neurons [26,27,28,29]. Specifically, glutamatergic neurons from the LHb mainly project to the RMTg [30,31], which in turn exerts a GABA-mediated inhibition onto VTA dopamine cells [26,32,33]. Nonetheless, some direct and reciprocal LHb–VTA projections are reported [34,35,36,37,38]. An increasing body of evidence over the last decade demonstrates that the influence of LHb–RMTg on the VTA is implicated in reward/aversion and positive/negative reinforcement induced by natural stimuli and drugs of abuse [26,28,30,36,38,39,40,41,42,43,44,45,46].

Consistent with their postulated role as the main components of the so-called brain anti-reward system, both the LHb and RMTg have emerged as critical players in the pathophysiology of depression and addiction [36,45,47,48,49,50,51]. In particular, recent behavioral and functional studies provide substantial support for the idea that multifaceted adaptations within these nuclei might be characterized, and causally linked to, the emergence of a withdrawal-induced aversive state in different animal models of drug dependence (i.e., alcohol, cocaine, and opiates) [48,50,52,53,54]. On the other hand, at present there is no evidence for whether and how the habenulo–mesencephalic pathway is affected during cannabis withdrawal. To address this issue, we investigated the effects of acute withdrawal after chronic THC administration on the LHb–RMTg–VTA circuit by using in vivo electrophysiological techniques. Our findings indicate that cannabinoid exposure and subsequent withdrawal profoundly alter the balance between inputs encoding rewarding and aversive signals on dopamine neurons.

## 2. Materials and Methods

### 2.1. Animals

Adult male Sprague–Dawley rats (Harlan Nossan, San Pietro al Natisone, Italy) weighing 250–400 g were housed in groups of four to six under standard conditions of temperature and humidity under a 12 h light/dark cycle with food and water available ad libitum. All experiments were conducted within the animal’s light cycle. All efforts were made to minimize animal suffering and to reduce the number of rats used.

### 2.2. Drugs

THC was purchased from THC PHARM GmbH (Frankfurt, Germany), rimonabant was a generous gift from Sanofi Research (Montepellier, France), and all other chemical compounds were purchased from Sigma-Aldrich. THC resin (cat.#17085) was dissolved in ethanol (cat.#459844) at 20% final concentration and then sonicated for 30 min. Both THC and rimonabant were emulsified in 1–2% Tween 80 (cat.#P1754) and then dissolved in sterile physiological saline (0.9% NaCl; cat.#S9625). Drugs were administered intraperitoneally (i.p.) or intravenously (i.v.) in volumes of 3 mL/kg and 1 mL/kg, respectively.

### 2.3. THC Chronic Administration

**Figure 1:**
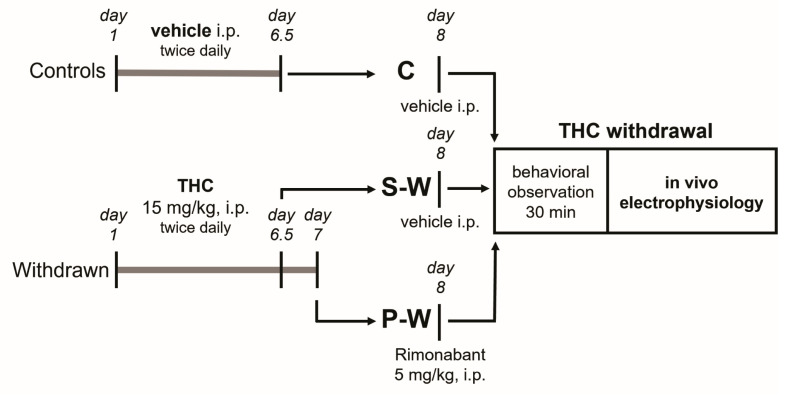
Diagram illustrating the experimental timeline. From day 1 to day 6.5 (C and S-W groups) or 7 (P-W group), rats received THC/vehicle, twice daily, i.p. On day 8, animals were challenged with vehicle (24 h after the last vehicle/THC administration; C and S-W groups) or with rimonabant (12 h after last THC administration; P-W group), then they underwent behavioral observation and in vivo electrophysiology.

The rats were randomly assigned to the experimental groups. Animals designated to the withdrawal condition were chronically treated with 15 mg/kg THC i.p. twice daily for 6.5 days (spontaneous withdrawal group, S-W) or 7 days (precipitated withdrawal group, P-W). Control group (C) rats received the vehicle. On day 8 from the onset of THC treatment, the P-W group received the CB_1_ receptor antagonist rimonabant (5 mg/kg i.p.). Behavioral observation and in vivo electrophysiology experiments were performed 12 or 24 h after the last administration (precipitated and spontaneous withdrawal, respectively) during the acute phase of withdrawal (Figure 1).

### 2.4. Behavioral Evaluation

For behavioral observation, on day 8, rats were individually placed in plexiglass cages (30 × 25 × 45 cm) with standard litter on the floor, located in a sound-proof room. Animals received rimonabant (P-W group) or vehicle (S-W and C groups), and after 10 min of habituation, cannabinoid withdrawal signs (i.e., licking, tongue rolling, facial rubbing, head shakes, wet dog shakes, scratching, forepaw fluttering, and paw treading) were scored by counting the total number of events over a 30 min period of time [18]. Point scoring was performed by an observer blind to the treatment.

### 2.5. In Vivo Electrophysiology

Rats were anesthetized with urethane (1.3 g/kg, i.p.) and their femoral veins were cannulated for i.v. administration of pharmacological agents. Animals were then placed in the stereotaxic apparatus (Kopf, Tujunga, CA, USA) with their body temperature maintained at 37 ± 1 °C by a heating pad. The scalp was retracted, and one burr hole was drilled above the area selected for the experiments, according to the stereotaxic rat brain atlas of Paxinos and Watson [55]. Single-unit activity of neurons was extracellularly recorded with glass micropipettes filled with 2% pontamine sky blue (PSB) dissolved in 0.5 M sodium acetate (3–5 MΩ impedance). Individual action potentials were amplified (Neurolog System, Digitimer, Hertfordshire, UK) and displayed on a digital storage oscilloscope (TDS 3012, Tektronics, Marlow, UK). The experiments were sampled online with Spike2 7.20 software by a computer connected to the CED 1401 interface (Cambridge Electronic Design, Cambridge, UK).

To estimate the cell population’s spontaneous activity, the electrode was passed within each brain area in 4–6 predetermined tracks separated by 200 μm and the total number of active cells encountered in each brain area was divided by the number of tracks (cells/track). At the end of recording sessions, DC current (15 mA for 20 min) was passed through the recording micropipette to eject PSB for marking the recording site. Then, the animals were euthanized with a lethal dose of urethane, and their brains were rapidly removed and frozen in isopentane. The position of the electrodes was microscopically identified on serial 60 μm sections stained with neutral red. Only cells from subjects for which correct electrode placement was verified histologically were included in the study [56].

Experiments in the posterior VTA. We selected the medio-lateral portion of parabrachial pigmented nuclei (PBP; AP, 5.8–6.0 mm posterior to bregma; L, 0.4–0.6 mm from midline; V, 7.0–8.0 mm below cortical surface), which has been shown to contain a larger density of dopamine neurons when compared to the more medial levels of the posterior VTA [57,58]. Single putative dopamine neurons were isolated (bandpass filter 0.1–10.000 Hz) and identified according to firing rate (<10 Hz) and duration of action potential (>2.5 ms), as measured from start to end [26]. Bursts were defined as the occurrence of at least two spikes at an inter-spike interval (ISI) of <80 ms and terminated when the ISI exceeded 160 ms [59,60]. To evaluate the inhibitory input arising from the RMTg to the VTA, a formvar-coated stimulating stainless steel bipolar electrode (250 μm tip diameter) was aimed at the ipsilateral RMTg. The electrode was inserted with an inclination of 20° anteroposterior on the coronal plane (AP, 9.6 mm posterior to bregma; L, 0.8 mm from midline; V, 7.0 mm below cerebellar surface). Once a cell was selected, electrical stimuli consisting of single, monophasic, rectangular pulses (0.5 mA, 0.3–0.5 ms) were delivered to the RMTg at 1 Hz. The responses to electrical stimulation of the RMTg were evaluated, and a peristimulus time histogram (PSTH) was generated off-line for each neuron. A cell was classified as inhibited or excited when the number of action potentials/bin (bin size = 1 ms) in the 50 ms after the stimuli was significantly lower or higher, respectively, than baseline levels. The duration of stimulus-evoked inhibition was defined as the time of complete cessation of firing after the stimuli [56].

Experiments in the RMTg. Within the RMTg (AP, 7.0–7.4 mm posterior to bregma; L, 0.7–0.8 mm from midline; V, 6.5–7.5 mm below cortical surface), putative GABA neurons were isolated (bandpass filter 1–3.000 Hz) and identified according to previously described electrophysiological characteristics, including a relatively high spontaneous firing rate (>10 Hz) and a biphasic and short (<1.5 ms) action potential [30,42]. To identify RMTg neurons receiving inputs from the LHb, a formvar-coated stainless steel bipolar electrode (250 μm tip diameter) was inserted in the ipsilateral LHb (AP, 1.9 mm posterior to bregma; L, 0.7 mm lateral to the midline; V, 4.7 mm below cerebral surface) with an inclination of 20° anteroposterior on the coronal plane. Once a cell was selected, 0.5 mA stimuli were delivered to the LHb at 1 Hz. Excitatory responses of RMTg cells to electrical stimulation of the LHb were evaluated and a PSTH was generated off-line for each neuron.

Experiments in the LHb. Within the LHb (AP, 3.4–3.6 mm posterior to bregma, L, 0.7–0.8 mm from midline, V, 4.5–6.0 mm below cortical surface), putative glutamate neurons were isolated (bandpass filter 1–3.000 Hz) and identified using previously described electrophysiological criteria, including a triphasic and broad (>3.0 ms) action potential and a mean firing rate of about 14 Hz [61,62]. Bursts were defined as the occurrence of at least two spikes at ISI <20 ms and terminated when the ISI exceeded 40 ms. To identify LHb neurons projecting to the RMTg, a stimulating electrode was inserted in the ipsilateral RMTg with an inclination of 20° anteroposterior on the coronal plane (AP, 9.6 mm posterior to bregma; L, 0.8 mm from midline; V, 7.0 mm below cerebellar surface). Once an LHb cell was selected, stimuli (0.5 mA) were delivered to the RMTg at 1 Hz. The criteria for antidromic response included the occurrence of one antidromic spike per stimulus with a constant latency (<1 msec drift) and possible collisions between elicited and spontaneously occurring spikes [63]. Antidromic responses to electrical stimulation of the RMTg were evaluated on-line and a PSTH was generated off-line for each cell, for confirmation.

### 2.6. Data Analysis and Statistics

Autocorrelograms using a 10 ms bin width for intervals of up to 2 s were generated for qualitative classification of the neuronal firing pattern as regular, irregular, or bursting. Autocorrelograms showing three or more regularly occurring peaks were characteristic of a regular firing pattern. An initial trough that rose smoothly to a steady state was classified as irregular, whereas an initial peak, followed by decay to a steady state, was typical of the bursting mode. The coefficient of variation (CV), a measure of action potential firing regularity, was determined as the standard deviation of ISIs divided by the mean ISI. The ISI histogram, a distribution of the time between subsequent action potentials, is an index of the neuronal pattern of discharge. ISI histograms were generated using a 10 ms bin width. Finally, the ISI distributions were obtained as a plot of averaged normalized frequencies for each group.

Statistical analysis was performed using Graphpad Prism 7 software (La Jolla, CA, USA). Data were analyzed using one-way or two-way ANOVA or the chi-square test for population comparisons when appropriate. Post hoc multiple comparisons were made using the Dunnett or Sidak test. THC-induced changes in duration of inhibition and firing rate were calculated by averaging the effects after THC administration (acute i.v. bolus injection) and normalizing to the pre-drug basal activity. All of the numerical data are given as the mean or percentage ± SEM. The significance level was established at *p* < 0.05.

## 3. Results

### 3.1. Behavioral Manifestations of THC Withdrawal

**Figure 2:**
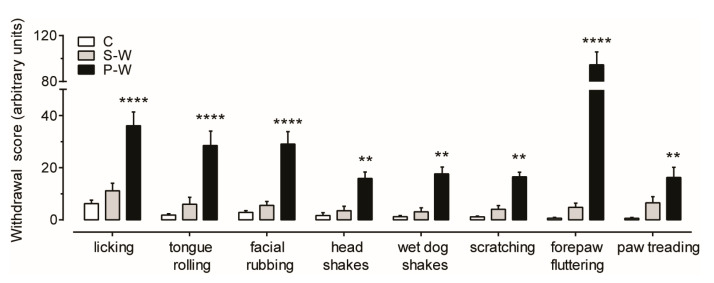
Behavioral manifestations of THC withdrawal. Bar histogram showing mean ± SEM of behavioral cannabinoid-withdrawal scores observed for 30 min for each group. P-W rats show a marked behavioral withdrawal syndrome (C, *n* = 16; S-W, *n* = 17; P-W, *n* = 16; two-way ANOVA and Sidak test, **** *p* < 0.0001, ** *p* < 0.01).

To evaluate behavioral manifestations of acute cannabinoid withdrawal, rats were observed 12–24 h after the last THC/vehicle administration (see Figure 1). We confirmed previous observations that THC withdrawal, precipitated by administration of the CB_1_ receptor antagonist rimonabant (P-W group), induced an intense behavioral manifestation characterized by a significant increase in some typical cannabinoid withdrawal signs such as scratching, facial rubbing, licking, and wet dog shakes [18]. Moreover, we extended our analysis and found that tongue rolling, head shakes, forepaw fluttering, and paw treading were also elevated in P-W rats (Figure 2). On the other hand, the spontaneous withdrawal (S-W) and control (C) group scores were not statistically different (S-W, *n* = 17, P-W, *n* = 16, C, *n* = 16; two-way ANOVA and Sidak test, F_(2,368)_ = 186.0; **** *p* < 0.0001, ** *p* < 0.01), thus corroborating the notion that spontaneous THC withdrawal does not induce overt phenotypic alterations.

### 3.2. The Hypodopaminergic State Underlying THC Withdrawal Is Concurrent with a Prolonged Duration of Inhibition Evoked by RMTg

**Figure 3:**
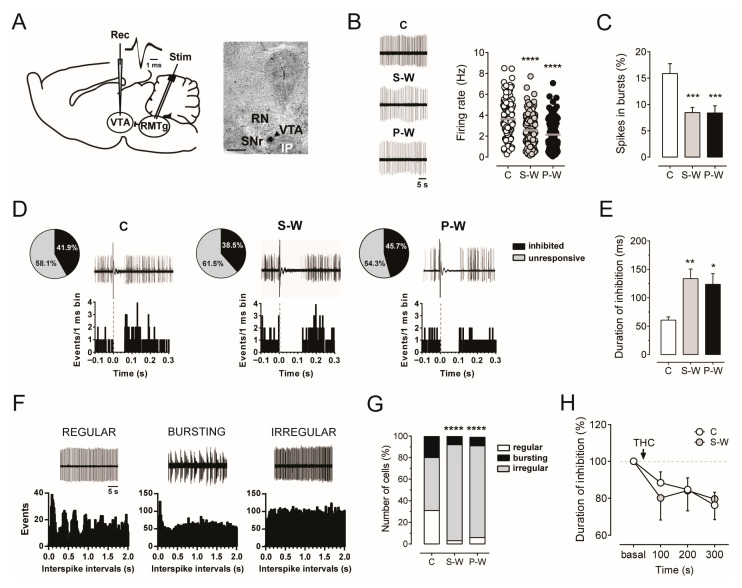
(**A**) On the left, schematic experimental protocol for in vivo electrophysiology recordings within the VTA (rec) and electrical stimulation of the RMTg (stim). Trace showing typical dopamine neuron action potential waveforms. On the right, recording site marked by the PSB dye (arrowhead) in a brain slice. Abbreviations: RN, red nucleus; IP, interpeduncular nucleus; SNr, substantia nigra pars reticulata; PBP, parabrachial pigmented nuclei. (**B**) Representative extracellular recordings of putative dopamine neurons in the VTA for each experimental group (left). Graphs showing the mean firing rate (right) and (**C**) the mean bursting activity (C, *n* = 13 rats, 125 cells; S-W, *n* = 15 rats, 167 cells; P-W, *n* = 11 rats, 109 cells). (**D**) Pie charts illustrating the percentages of inhibited (black) or unresponsive (grey) dopamine neurons following stimulation of the RMTg. Traces acquired from a digital oscilloscope [top] and PSTH of the same cell [bottom] showing that the duration of inhibition is increased in dopamine neurons from S-W (126 ms) and P-W (102 ms) rats when compared with controls (61 ms). (**E**) The bar graph represents the mean inhibitory response to RMTg stimulation in VTA dopamine cells from C (*n* = 49), S-W (*n* = 62), and P-W (*n* = 48) rats. The complete suppression of discharge activity in S-W and P-W rats was significantly longer than in C animals. (**F**) Representative extracellular recordings and relative autocorrelograms of regularly, bursting, and irregularly firing cells. (**G**) The bar graph shows that the number of irregularly firing neurons in S-W and P-W rats was increased. (**H**) Time course of acute THC’s effect on RMTg-induced inhibition of VTA dopamine neurons in C (*n* = 10) and S-W (*n* = 8) groups. The decrease in the duration of inhibition is reduced when compared to the baseline prior THC administration in both C and S-W rats. Data are expressed as mean or percentage of baseline ± SEM. One-way or two-way ANOVA for repeated measures followed by Dunnett test, Sidak test, or chi-square test when appropriate. **** *p* < 0.0001, *** *p* < 0.001, ** *p* < 0.01, * *p* < 0.05.

Extracellular single-unit recordings were performed for the three experimental groups (S-W, *n* = 15; P-W, *n* = 11; C, *n* = 13) to carry out population sampling of putative dopamine neurons (hereafter indicated as dopamine neurons). Cells in the lateral posterior VTA were identified as dopamine cells on the basis of their action potential characteristics, firing properties, and location within the PBP, a VTA sub-region that contains a large density of NAc-projecting tyrosine hydroxylase (TH)-positive neurons [35,64] (Figure 3A). In line with our previous report [18], we observed that the average firing rate (2.56 ± 0.11 Hz, *n* = 167, and 2.15 ± 0.15 Hz, *n* = 109, in S-W and P-W rats, respectively) as well as the percentage of burst firing (8.45 ± 0.98%, *n* = 167, and 8.39 ± 1.35%, *n* = 109, in S-W and P-W rats, respectively) were markedly decreased in dopamine cells recorded from withdrawn animals as compared with controls (firing rate: 3.63 ± 0.16 Hz, *n* = 125, F_(2,398)_ = 27.84; spikes in bursts: 15.86 ± 1.88%, *n* = 125, F_(2,398)_ = 9.204, one-way ANOVA and Dunnett test; **** *p* < 0.0001, *** *p* < 0.001, Figure 3B,C). Importantly, the cells/track index were not changed (1.78 ± 0.14, *n* = 15, 1.88 ± 0.18, *n* = 11, 1.54 ± 0.18, *n* = 13, in S-W, P-W, and C rats, respectively, one-way ANOVA and Dunnett test, *p* > 0.05), suggesting that the treatment did not affect the number of spontaneously active neurons. Together with the results above, these findings confirmed that both spontaneous and precipitated acute withdrawal from chronic THC treatment results in a reduction in dopamine neuronal functioning.

We formerly reported that, in naïve rats, the spontaneous discharge activity of VTA dopamine neurons was negatively correlated with the duration of RMTg-evoked inhibition and that the complete, though temporary, suppression of VTA dopamine firing elicited by RMTg electrical stimulation can be modulated by different drugs of abuse, including cannabinoids [26,42,65]. Accordingly, GABA inputs from the RMTg emerged as a major inhibitory cannabinoid-sensitive afferent regulating midbrain dopamine cell [26,29,32,40,42,66]. We therefore stimulated the RMTg during acute THC withdrawal while recording spontaneous VTA dopamine cell activity (Figure 3A). As expected, we found a significantly longer duration of RMTg-evoked inhibition in withdrawn rats as compared to controls (133.50 ± 16.98 ms, *n* = 62, and 123.60 ± 18.73 ms, *n* = 48, 60.55 ± 5.59 ms, *n* = 49, in S-W, P-W, and C rats, respectively; one-way ANOVA and Dunnett test, F_(2,156)_ = 6.480; ** *p* < 0.01, * *p* < 0.05; Figure 3D,E), with no differences in the percentage of inhibited cells among groups (71 out of 151, 38.5%, 48 out of 105, 45.7%, and 46 out of 110, 41.9%, in S-W, P-W, and C rats, respectively, chi-square test, *p* > 0.05; Figure 3D). These data support the hypothesis that enhanced GABA inputs from the RMTg might contribute to the hypodopaminergic state induced by acute THC withdrawal.

In addition, given that irregularly firing dopamine neurons exhibit a longer duration of inhibition in drug-naïve rats than regularly or bursting ones [26], we further classified RMTg-inhibited dopamine neurons according to their pattern of discharge (Figure 3F). In agreement with the observed prolonged suppression of VTA dopamine cell discharge in both S-W and P-W rats (Figure 3E), we found an increased number of dopamine neurons that fired irregularly in the same groups (55 out of 62, 89%, 41 out of 48, 85%, and 24 out of 49, 49%, in S-W, P-W, and C rats, respectively, chi-square test, **** *p* < 0.0001; Figure 3G). These findings suggested that the changes in the pattern of discharge during THC withdrawal might be responsible for the increased inhibition of dopamine neurons induced by RMTg projections.

We previously demonstrated that the duration of VTA dopamine cell inhibition evoked by stimulation of the RMTg was reduced by the synthetic CB_1_ receptor agonist WIN55212-2 (WIN) in naïve rats [26], and that RMTg axon terminals impinging upon VTA dopamine neurons expressed CB_1_ receptors [65]. Hence, we investigated the effect of CB_1_ receptor activation on RMTg-induced suppression of VTA dopamine cell activity during spontaneous THC withdrawal. We found that acute intravenous administration of THC (1.2 mg/kg) reduced the duration of inhibition over time in both C (*n* = 10) and S-W rats (*n* = 8, two-way ANOVA for repeated measures, interaction time × treatment F_(3,48)_ = 0.4026, *p* = 0.7518, time F_(2.428,38.85)_ = 5.950, *p* = 0.0035; Figure 3H), indicating that the RMTg-mediated control of VTA dopamine neuron firing is not susceptible to tolerance following repeated THC exposure and subsequent withdrawal.

### 3.3. Decline of RMTg Neuron Spontaneous Activity during THC Withdrawal

**Figure 4:**
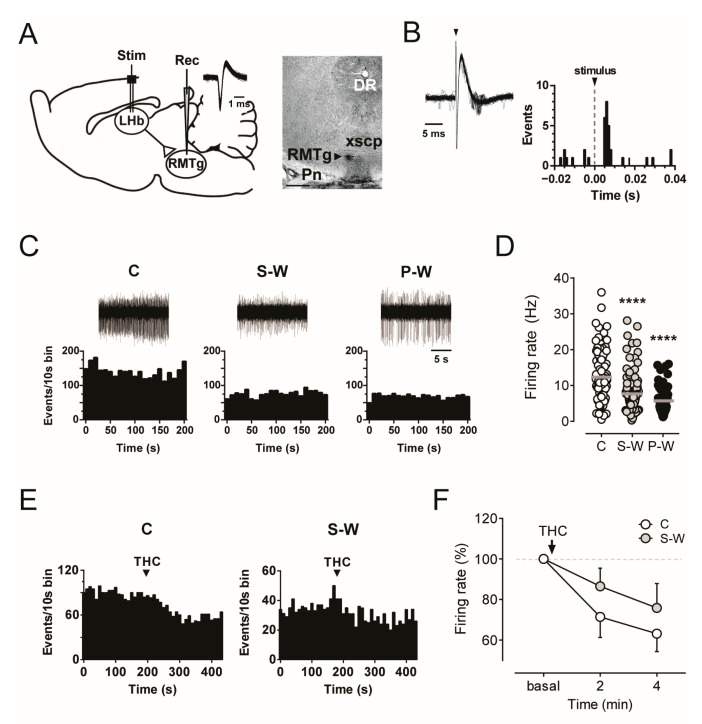
(**A**) On the left, schematic illustration of the experimental protocol for in vivo electrophysiological recordings in the RMTg (rec) and stimulation in the LHb (stim). Trace showing superimposed RMTg neuron action potential waveforms. On the right, recording location for the RMTg marked by the PSB dye (arrowhead) in a brain slice. Abbreviations: Pn, pontine nuclei; xscp, decussation of the superior cerebellar peduncle; DR, dorsal raphe. (**B**) Superimposed traces acquired from a digital oscilloscope showing a relatively constant latency of the orthodromic response of RMTg neuron following LHb stimulation (left) and representative PSTH (right). (**C**) Recording trace [top] and rate histogram (bottom) of single RMTg neuron encountered in rats belonging to the C, S-W, and P-W groups. (**D**) Graph showing the mean firing rate of RMTg cells (C, *n* = 9 rats, 87 cells; S-W, *n* = 9 rats, 104 cells; P-W, *n* = 7 rats, 69 cells). (**E**) Rate histograms showing that the THC-induced reduction in the firing activity of RMTg neurons from C rats is absent in RMTg neurons from S-W rats. (**F**) Graph showing averaged time course of THC-induced firing rate reduction (C, *n* = 8; S-W, *n* = 7). Data are expressed as mean or percentage of baseline ± SEM. One-way or two-way ANOVA for repeated measures followed by Dunnett test or Sidak test when appropriate. **** *p* < 0.0001.

The finding of a longer duration of inhibition induced by RMTg stimulation on VTA dopamine neurons prompted us to analyze the spontaneous electrical activity of RMTg putative GABA neurons (hereafter, GABA neurons) in THC withdrawn animals. We performed single-unit extracellular recordings from the RMTg region of withdrawn anesthetized rats (S-W, *n* = 9; P-W, *n* = 7; C, *n* = 9; Figure 4A). RMTg GABA cells were identified by their biphasic and narrow (<1.5 ms) action potential waveform, relatively high spontaneous firing rate (average >10 Hz). Moreover, we tested the excitatory response at short latency (<8 ms) evoked by stimulation of the LHb, as previously described [26,32,42] (Figure 4A,B).

When we compared the basal discharge rates of RMTg GABA neurons among the experimental groups, our data revealed a marked decrease in both S-W and P-W rats (7.56 ± 0.54 Hz, *n* = 104, and 5.72 ± 0.57 Hz, *n* = 69, in S-W and P-W groups, respectively) with respect to C subjects (12.36 ± 0.80 Hz, *n* = 87, one-way ANOVA and Dunnett test, F_(2,257)_ = 25.67; **** *p* < 0.0001; Figure 4C,D), with no changes in the number of spontaneously active cells (2.76 ± 0.23, *n* = 9, 2.35 ± 0.42, *n* = 7, 2.42 ± 0.25, *n* = 9, in S-W, P-W, and C rats, respectively, one-way ANOVA and Dunnett test, *p* > 0.05). Hence, THC withdrawal increases RMTg to VTA presynaptic inhibitory inputs independently of RMTg neuron somatic firing.

We next tested whether RMTg GABA neurons showed tolerance to the acute effect of THC during spontaneous withdrawal. Our analysis revealed that THC (1.2 mg/kg, i.v.) produced a decrease in the RMTg neuron firing rate over time in both C (*n* = 8) and S-W rats (*n* = 7, two-way ANOVA for repeated measures, interaction time x treatment F_(2,30)_ = 0.8298, *p* = 0.4459, time F_(1.431,21.47)_ = 12.26, *p* = 0.0008; Figure 4E,F). Thus, our results confirmed that acute administration of cannabinoids reduces RMTg frequency of discharge in drug-naïve animals [42], while no apparent tolerance to THC-induced inhibition was observed in the RMTg cells of S-W rats.

### 3.4. Functional Alterations of LHb Neurons During Acute THC Withdrawal

The LHb controls the midbrain areas and is critically involved in regulating negative emotional states associated with mood and substance use disorders [36,44,45,48,50]. Hence, we next assessed whether LHb glutamate neuron dysfunctions also occurred during acute THC withdrawal.

**Figure 5:**
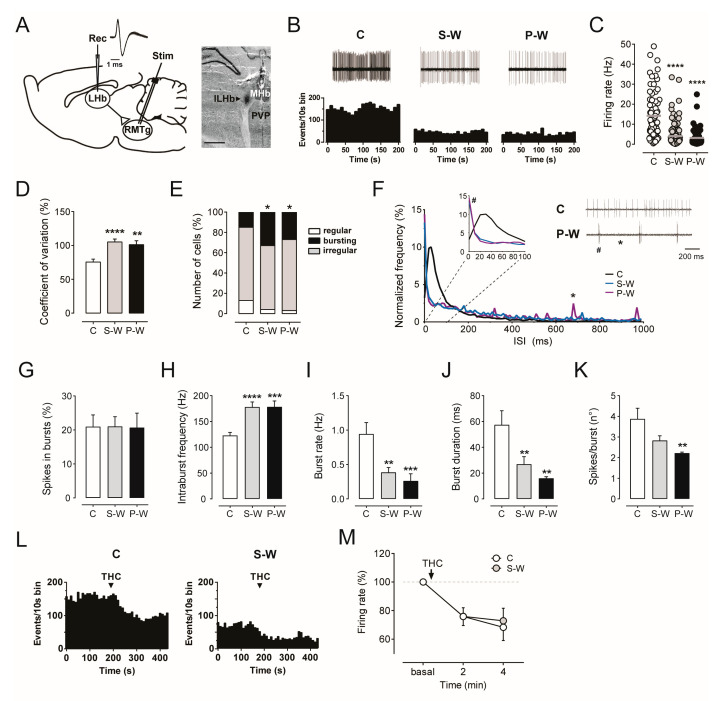
(**A**) On the left, diagram illustrating the experimental protocol for in vivo electrophysiological recordings in the LHb (rec) and stimulation in the RMTg (stim). Trace showing superimposed LHb neuron action potential waveforms. On the right, recording site for the LHb marked by the PSB dye (arrowhead) in a brain slice. Abbreviations: lLHb, lateral subdivision of LHb; MHb, medial habenula; PVP, posterior paraventricular thalamus. (**B**) Spontaneous activity of LHb neurons encountered in a rat belonging to the C, S-W, and P-W groups [top]. Each rate histogram (bottom) represents the neuronal activity of a single neuron. Graphs showing the mean firing rate (**C**) and the mean percentage of CV (**D**) (C, *n* = 9 rats, 72 cells; S-W, *n* = 10 rats, 107 cells; P-W, *n* = 4 rats, 58 cells). (**E**) The bar graph shows that the number of bursting neurons in S-W and P-W rats is increased. (**F**) Graph displaying averaged normalized frequency of inter-spike intervals (ISI) from LHb neurons recorded from C, S-W, and P-W rats. The enlargement of the first 100 ms shows that LHb cells from withdrawn animals fire with shorter inter-spike intervals, as expected from burst firing cells. On the right, example traces from C and P-W rats illustrate dissimilar intervals among action potentials. In P-W rats, the # points at intervals among spikes within the burst (~10 ms), the * points at intervals between bursts (~700 ms). Graphs showing the mean percentage of spikes in bursts (**G**) (C, *n* = 9 rats, 72 cells; S-W, *n* = 10 rats, 107 cells; P-W, *n* = 4 rats, 58 cells), the mean intraburst frequency (**H**), the mean burst rate (**I**), the mean burst duration (**J**), and the mean number of spikes per burst (**K**) (C, *n* = 9 rats, 64 cells; S-W, *n* = 10 rats, 69 cells; P-W, *n* = 4 rats, 39 cells). (**L**) Rate histogram showing the decreased firing rate induced by THC in LHb neurons from C and S-W rats. (**M**) Averaged time course of firing rate decrease following THC administration (C, *n* = 9; S-W, *n* = 7). Data are expressed as mean or percentage of baseline ± SEM. One-way or two-way ANOVA for repeated measures followed by Dunnett test, Sidak test, or chi-square test when appropriate. **** *p* < 0.0001, *** *p* < 0.001, ** *p* < 0.01, * *p* < 0.05.

To accomplish this aim, extracellular single-unit recordings from LHb putative glutamate neurons (hereafter glutamate neurons) were carried out in withdrawn anesthetized rats (S-W, *n* = 10; P-W, *n* = 4; C, *n* = 9; Figure 5A). In line with previous reports [61,62], we selected LHb neurons based on their large waveform (>3 ms) and high basal firing rate (average >15 Hz). The antidromic response at short latency (<10 ms) evoked by stimulation of the RMTg was also recorded.

Consistent with the above described depression of RMTg electrical activity, we found that the average frequency of LHb neurons was strongly reduced in withdrawn rats (4.08 ± 0.59 Hz, *n* = 107, and 3.12 ± 0.60 Hz, *n* = 58, in S-W and P-W groups, respectively) as compared to C rats (14.21 ± 1.37 Hz, *n* = 72, one-way ANOVA and Dunnett test, F_(2,234)_ = 43.77; **** *p* < 0.0001; Figure 5B,C). In addition, the analysis of the coefficient of variation (CV) revealed more irregular firing activity during both THC S-W and P-W conditions (105.7 ± 4.4%, *n* = 107, and 101.2 ± 5.7%, *n* = 58, 75.6 ± 4.2%, *n* = 72, in S-W, P-W, and C groups, respectively, one-way ANOVA and Dunnett test, F_(2,234)_ = 11.72; **** *p* < 0.0001, ** *p* < 0.01; Figure 5D). The cells/track index did not differ among groups (3.24 ± 0.39, *n* = 10, 3.41 ± 0.66, *n* = 4, 3.07 ± 0.37, *n* = 9, in S-W, P-W, and C rats, respectively, one-way ANOVA and Dunnett test *p* > 0.05). To classify the pattern of discharge of LHb neurons as regular, bursting, or irregular, we then analyzed their autocorrelograms. Interestingly, the proportion of bursting cells was augmented in S-W and P-W rats with respect to C rats (36 out of 107, 34%, 16 out of 58, 28%, and 11 out of 72, 15%, in S-W, P-W, and C rats, respectively, chi-square test, * *p* < 0.05; Figure 5E). Accordingly, the plot of inter-spike intervals (ISI) displayed a higher frequency of short ISI in LHb cells from withdrawn rats (Figure 5F). Furthermore, the analysis of bursting activity revealed that LHb neurons did not show differences in the % of spikes in bursts among groups (21.01 ± 2.88%, *n* = 107, 20.69 ± 4.25%, *n* = 58, 20.95 ± 3.46%, *n* = 72, in S-W, P-W, and C rats, respectively, one-way ANOVA and Dunnett test *p* > 0.05; Figure 5G).

On the other hand, in LHb cells from withdrawal groups, we observed a significant increase in the intraburst frequency (178.0 ± 9.85 Hz, *n* = 69, 178.4 ± 11.39 Hz, *n* = 39, 122.9 ± 5.85 Hz, *n* = 64, in S-W, P-W, and C rats, respectively, one-way ANOVA and Dunnett test, F_(2,169)_ = 13.18, **** *p* < 0.0001, *** *p* < 0.001; Figure 5H), a lower burst rate (0.39 ± 0.07 Hz, *n* = 69, 0.26 ± 0.10 Hz, *n* = 39, 0.94 ± 0.17 Hz, *n* = 64, in S-W, P-W, and C rats, respectively, one-way ANOVA and Dunnett test, F_(2,169)_ = 8.477, *** *p* < 0.001, ** *p* < 0.01; Figure 5I), and a shorter burst duration (26.88 ± 5.92 ms, *n* = 69, 15.98 ± 1.25 ms, *n* = 39, 57.38 ± 10.98 ms, *n* = 64, in S-W, P-W, and C rats, respectively, one-way ANOVA and Dunnett test, F_(2,169)_ = 6.55, ** *p* < 0.01; Figure 5J). Moreover, in P-W rats, LHb neurons showed a significant decrease in the number of spikes per burst (2.83 ± 0.23, *n* = 69, 2.22 ± 0.05, *n* = 39, 3.88 ± 0.52, *n* = 64, in S-W, P-W, and C rats, respectively, one-way ANOVA and Dunnett test, F_(2,169)_ = 4.696, ** *p* < 0.01; Figure 5K). Overall, our results indicated that acute THC withdrawal reduces the LHb tonic firing rate, whereas it increases the proportion of cells displaying phasic activity.

Finally, we studied the effect of acute THC administration on LHb neurons. THC (1.2 mg/kg, i.v.) induced an inhibited discharge frequency over time in both C (*n* = 9) and S-W rats (*n* = 7, two-way ANOVA for repeated measures, interaction time x treatment F_(2,28)_ = 0.1326, *p* = 0.8763, time F_(1.394,19.51)_ = 18.95, *p* = 0.0001; Figure 5L,M), thus supporting the presence of functional CB_1_ receptors within the LHb and the lack of THC tolerance after chronic exposure and withdrawal.

## 4. Discussion

In an animal model of cannabinoid withdrawal, we analyzed in vivo electrophysiological properties of single neurons from brain areas processing reward and aversion, such as the VTA, the RMTg, and the LHb. We confirmed our previous finding that withdrawal following protracted THC exposure induces hypoactivity of VTA dopamine neurons [18], as shown by the reduced firing rate and percentage of spikes in burst, with no changes in the number of spontaneously active cells. Though inducing spontaneous withdrawal may be prone to false negatives arising from slow elimination of THC and its metabolites [67], it represents a translational model for human cannabis withdrawal [68]. The hypodopaminergic tone was shown by both spontaneous and precipitated groups, nonetheless it did not correlate with somatic manifestations exclusively induced by pharmacologically precipitated withdrawal, thus confirming (scratching, facial rubbing, licking, and wet dog shakes) and extending (tongue rolling, head shakes, forepaw fluttering, and paw treading) our previous report [18].

By electrophysiology, we further showed that the inhibitory input from the RMTg, a major GABA projection to the VTA, was strengthened. The RMTg–VTA pathway is known to process the acute effects of cannabinoids, but its role in other stages of CUD was neglected. Indeed, we previously demonstrated the presence of CB_1_ receptors in RMTg terminals into the VTA [65], confirming disinhibition of dopamine neurons as a mechanism for the rewarding effect following acute cannabinoid exposure. Consistently, administration of the CB_1_ agonist WIN55,212-2 decreased the firing rate of RMTg neurons and reduced the duration of inhibition imposed to VTA dopamine neurons [26]. Although cannabinoid withdrawal reduced the firing rate of RMTg cells, it prolonged the duration of dopamine neuron inhibition, indicating that the activity of their synaptic terminals was uncoupled from somatic firing. Our findings suggest local control of GABA inputs within the VTA by the RMTg, consistent with recent research showing that presynaptic CB_1_ receptor coupling to the neurotransmitter release machinery dictates synapse-specific release probability and strength, which are disrupted by in vivo THC exposure [69]. Chronic THC induces CB_1_ receptor downregulation in several brain areas, including the cerebral cortex, basal ganglia, limbic system, and hippocampus [70,71]. These mechanisms collectively might reduce the inhibitory control of GABA released from RMTg terminals.

Prolonged inhibition evoked by stimulation of RMTg is characteristic of irregularly firing dopamine cells. This effect, previously shown by our group after acute cannabinoid exposure [26], aligns with the current finding of an increased number of irregularly firing neurons in the VTA of THC-withdrawn rats, alongside a decreased number of neurons with regular or burst firing patterns. This suggests that chronic THC concurrently promotes the sensitization to GABA and alters the firing pattern, ultimately leading to a decreased dopaminergic tone.

In our model, the reduced electrophysiological activity of RMTg GABA neurons was to some extent unexpected. The RMTg is known to increase its function during aversive conditions such as noxious stimuli [72], thereby producing corresponding inhibition of dopamine neurons. However, the status of withdrawal represents a multifaceted condition, which does not correspond to mere aversion. Accordingly, the firing of RMTg neurons was found to be significantly decreased after morphine withdrawal [53], suggesting that common mechanisms could sustain abstinence from drugs with different target receptors.

A decrease in activity might also represent a counterbalance to the enhanced GABA tone and the resulting increased inhibition imposed on dopamine neurons. Alternatively, the RMTg might be receiving reduced excitatory input from projecting areas such as the LHb, which is involved in encoding aversive stimuli [32,38]. In rodents, CB_1_ receptors are expressed in the LHb by both pre- and postsynaptic neurons as well as glial cells [73,74]. Since the endocannabinoid system in this area is disrupted specifically by stressful conditions [73], examination of the LHb during cannabis withdrawal is crucial.

In line with these premises, we found that LHb neurons from withdrawn animals exhibited a decreased firing rate and altered pattern of activity, as shown by a different number of bursting cells and their parameters. Interestingly, acute THC administration further inhibited the firing rate both in C and SW rats, suggesting little tolerance to THC effects. The reduced glutamatergic tone from the LHb might ultimately contribute to the depressed firing activity in terminal areas, including the RMTg and the VTA [75]. In apparent contrast, our analysis of the LHb neuron pattern of activity revealed an increase in bursting cells in withdrawn rats associated with a higher intraburst frequency, which was suggestive of an enhancement in LHb neuron phasic activity. Nevertheless, thus far we cannot rule out the possibility that this phenomenon might represent a compensatory mechanism to counteract the dramatic depression in the firing rate of LHb neurons observed during THC withdrawal.

Over the last two decades, adaptations within the habenulo–mesencephalic circuit have emerged as a hallmark of drug withdrawal [48]. In accordance with our findings, early studies demonstrated that chronic exposure to cocaine and other psychostimulants leads to fasciculus retroflexus degeneration, mainly in the portion containing LHb axons projecting to the ventral mesencephalon, suggesting that continuous stimulant drug administration weakens the excitatory link that LHb glutamate cells send to VTA dopamine and RMTg GABA neurons [76,77]. On the other hand, more recent evidence points to glutamatergic and GABAergic synaptic modifications in the LHb, which lead to neuronal hyperactivity, as crucial mechanisms for the emergence of aversive effects during cocaine withdrawal (reviewed in [78] and in [48]).

For instance, it has been shown that in mice chronically treated with cocaine, LHb neurons projecting to the RMTg but not to the VTA exhibited synaptic potentiation ex vivo and an enhanced firing rate in vivo, which persisted until 14 days after the last cocaine administration [79]. A comparable scenario occurs during ethanol withdrawal in rats. After a 24 h withdrawal period from chronic administration of ethanol, LHb neurons display both increased membrane excitability and in vivo firing activity [80,81]. In addition, during acute ethanol withdrawal, the LHb is more active and the RMTg exhibits higher activity even though without the recruitment of LHb inputs [82]. However, while cocaine and ethanol withdrawal-mediated effects in the LHb are almost overlapping, i.e., complementary changes sustaining increased excitation of the LHb, the picture is different in the case of opioids (see 51 for a review). Indeed, Valentinova et al. [83] reported that withdrawal from prolonged morphine leads to a decrease in the AMPA ratio in LHb neurons projecting to the dorsal raphe nucleus. Interestingly, this plasticity was observed during naloxone-precipitated withdrawal and in spontaneous withdrawal, lasting up to 30 days after morphine suspension. Additionally, a similar reduction in the AMPA ratio in the LHb occurs after foot-shock stress, which is linked to maladaptive behaviors [84].

In this regard, the present results also align with observations that acute morphine withdrawal is associated with a higher probability of GABA release from the RMTg to the VTA due to upregulation of the adenylate cyclase cascade [85]. These findings emphasize the well-established parallels between opioids and cannabinoids, particularly their ability to stimulate dopamine neurons in the VTA after acute intake [26,42,66,86] and to induce hypodopaminergia over the course of withdrawal after prolonged administration [87,88]. Further investigation is needed to elucidate the role of the LHb and its endocannabinoid system in different substance use disorders.

## 5. Conclusions

Our study supports the hypothesis that the cannabinoid withdrawal-induced hypodopaminergic condition is sustained by altered neurotransmission at the terminal level, possibly resulting from the disruption of presynaptic CB_1_ receptors following prolonged THC exposure. These functional changes within the habenulo–mesencephalic circuit may contribute to different stages of cannabis and other substance use disorders in a terminal-specific manner, with potentially shared mechanisms that warrant further investigation. Understanding these alterations provides valuable insight into the neural adaptations underlying cannabis withdrawal and highlights potential targets for therapeutic intervention in substance use disorders.

## Data Availability

The raw data supporting the conclusions of this article will be made available by the authors upon request.
